# Vaso‐occlusive crisis and acute chest syndrome in sickle cell disease due to 2019 novel coronavirus disease (COVID‐19)

**DOI:** 10.1002/ajh.25821

**Published:** 2020-04-21

**Authors:** Erfan Nur, Aafke E. Gaartman, Charlotte F. J. van Tuijn, Man Wai Tang, Bart J. Biemond

**Affiliations:** ^1^ Department of Clinical Hematology Amsterdam University Medical Centers Amsterdam The Netherlands

In March 2020, during the 2019 novel coronavirus disease (COVID‐19) pandemic, caused by the newly emerged virus SARS‐CoV‐2, two patients with homozygous sickle cell disease (SCD) were admitted to our hospital with a painful vaso‐occlusive crisis (VOC) triggered by COVID‐19. Both patients had no flu‐like complaints characteristic of COVID‐19 during or preceding the VOC episode.

**IMAGE 1:**
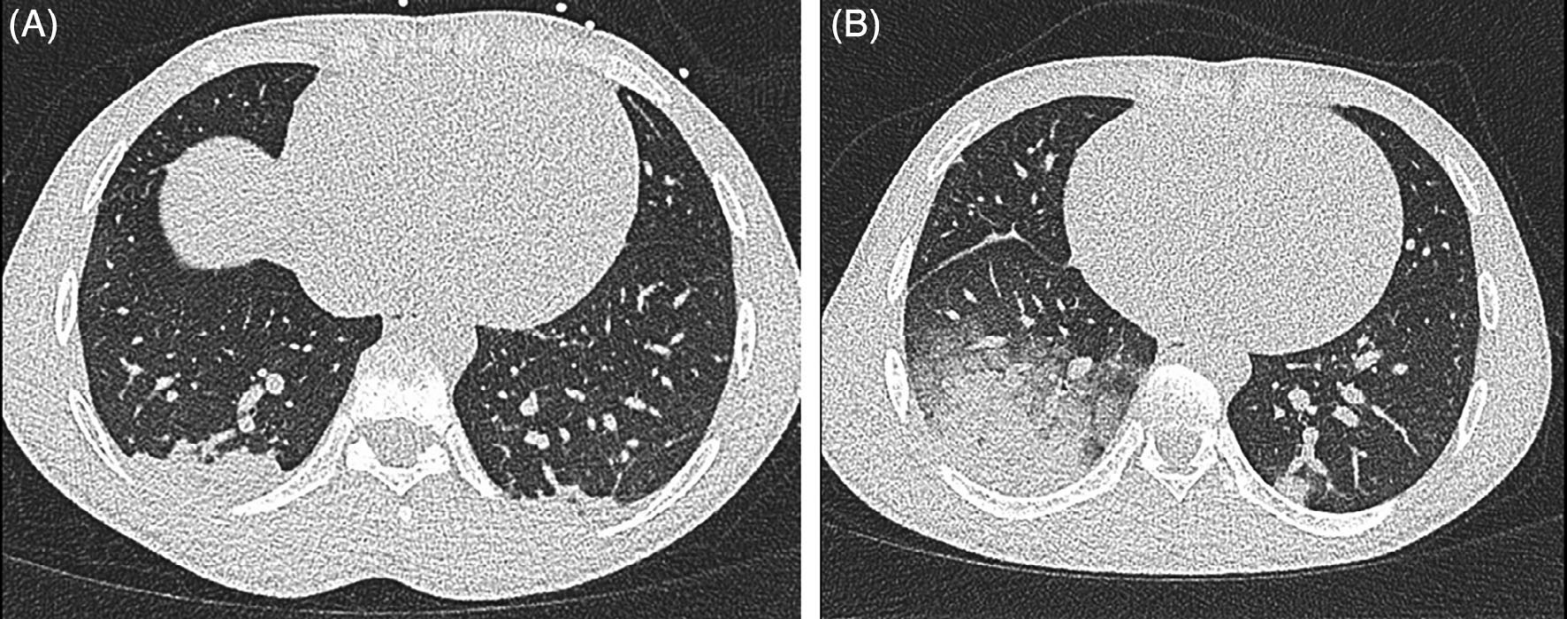
A, Chest CT imaging of patient 1 at first presentation to the emergency room (ER) showing infiltrates at basal fields. B, CT imaging at second ER presentation of patient 1, showing an increase in double‐sided infiltrates

Patient 1, a 24‐year‐old man with a previous medical history of minor pain episodes without indication for hospitalization, presented with severe right thoracic pain for 3 days. At presentation he had a temperature of 37.6°C; pulse of 76/minute, blood pressure of 106/65 mmHg; respiration rate of 18/min and a peripheral oxygen saturation (SpO_2_) of 97%. A non‐contrast chest CT showed double‐sided infiltrates without ground‐glass opacities or crazy paving and was not characteristic of COVID‐19 (Image 1A). Throat and nose swabs were negative for SARS‐CoV‐2.

A diagnosis of VOC complicated by acute chest syndrome (ACS) was made. Treatment with oxygen, intravenous morphine with patient‐controlled analgesia (PCA), fluid replacement therapy and amoxicillin/clavulanic acid was initiated. After 1 day, the level of pain had decreased significantly (numeric rating scale decreasing from nine to two) and the patient remained respiratory stable throughout his hospital stay. He was discharged with amoxicillin/clavulanic acid continued at home. However, the next day he returned to the emergency room with increased pain, dyspnea, respiration rate of 20/minutes, SpO_2_ of 93% and a temperature of 38.9°C. Chest CT imaging showed progression of the double‐sided infiltrates in the lower lobes of the lungs (Image 1B). A second PCR, this time of a sputum sample, was positive for SARS‐CoV‐2. He was treated with oxygen (up to 5 L/min) and morphine (PCA) while amoxicillin/clavulanic was continued. The patient had a smooth recovery, without a need for exchange transfusion. He was discharged again after 3 days and was instructed to stay at home (in isolation) until 24 hours after becoming completely symptom‐free.

Patient 2, a 20‐year‐old woman with a history of frequent VOCs presented with severe pain of the back and extremities for 1 day. She had no respiratory or gastro‐intestinal complaints. Because of a dip in SpO_2_ to 88% after a 100 μg parenteral fentanyl bolus in the ambulance, the suspicion of COVID‐19 rose which led to performing a chest CT and a throat and nose swab for a SARS‐CoV‐2 PCR. While the CT imaging did not show any pulmonary abnormalities, the PCR was positive for SARS‐CoV‐2. She remained hospitalized to treat her VOC without developing any respiratory symptoms.

The World Health Organization (WHO) recently declared SARS‐CoV‐2 infection a pandemic. Severe respiratory illness occurs in approximately 15%‐20% of infected patients.^1^ As of March 31, 2020, 800.049 laboratory‐confirmed cases and 38.714 deaths have been documented globally.^2^ In SCD, COVID‐19 can potentially cause severe (pulmonary) complications, either by directly causing severe pneumonia or by triggering a VOC and/or ACS. While further experience regarding the clinical presentation of COVID‐19 in SCD needs to be awaited, the following important points need to be taken into consideration based on the above described patients.

Similar to what we know from other viral infections,^3^, ^4^ SARS‐CoV‐2 can also cause ACS in SCD. Furthermore, as can be seen in patient 1, an ACS can develop without the typical pulmonary complications that can be seen with COVID‐19. Both patient 1 and especially patient 2 illustrate that COVID‐19 might trigger a VOC without the presence of flu‐like symptoms of COVID‐19. With respect to diagnosis, the history of patient 1 illustrates the low sensitivity of the PCR of the throat and nose swabs in the primary diagnosis of COVID‐19, which is estimated to be around 70%. We therefore suggest to perform a second PCR, preferably on a sputum sample, and non‐contrast chest CT imaging when there is no alternative explanation for VOC or when the clinical suspicion for COVID‐19 remains high.

Based on these two patients, at our center we decided to include SARS‐CoV‐2 PCR in the evaluation of SCD patients presenting with VOC.
